# Fine-tuning spermidine binding modes in the putrescine binding protein PotF

**DOI:** 10.1016/j.jbc.2021.101419

**Published:** 2021-11-19

**Authors:** Pascal Kröger, Sooruban Shanmugaratnam, Ulrike Scheib, Birte Höcker

**Affiliations:** 1 Department for Biochemistry, University of Bayreuth, Bayreuth, Germany; 2 Max Planck Institute for Developmental Biology, Tübingen, Germany

**Keywords:** periplasmic binding proteins, ligand specificity swap, protein engineering, adaptability, ITC, isothermal titration calorimetry, PBP, periplasmic binding protein, PDB, Protein Data Bank, PUT, putrescine, SPD, spermidine

## Abstract

A profound understanding of the molecular interactions between receptors and ligands is important throughout diverse research, such as protein design, drug discovery, or neuroscience. What determines specificity and how do proteins discriminate against similar ligands? In this study, we analyzed factors that determine binding in two homologs belonging to the well-known superfamily of periplasmic binding proteins, PotF and PotD. Building on a previously designed construct, modes of polyamine binding were swapped. This change of specificity was approached by analyzing local differences in the binding pocket as well as overall conformational changes in the protein. Throughout the study, protein variants were generated and characterized structurally and thermodynamically, leading to a specificity swap and improvement in affinity. This dataset not only enriches our knowledge applicable to rational protein design but also our results can further lay groundwork for engineering of specific biosensors as well as help to explain the adaptability of pathogenic bacteria.

Biomolecular recognition and discrimination are crucial for many biological functions. Binding interactions ranging from highly specific to promiscuous determine the regulation and functioning of a multitude of parallel cellular processes (1, 2). Periplasmic binding proteins (PBPs) are versatile bacterial nonenzymatic receptors that sense a range of different solutes, like carbohydrates, amino acids, vitamins, and ions (3). This large ligand diversity is reflected in a high sequence diversity within the superfamily, although the general structure of PBPs is conserved (4). They consist of two β/α-lobes connected *via* a hinge region with the ligand-binding site located at the interface of the lobes. PBPs are predominantly open in solution and close by undergoing a large conformational change upon ligand recognition, which is often compared to a Venus flytrap (5). These receptors work hand in hand with bacterial prokaryotic-type ABC transporters, since they require additional solute-binding proteins to recruit substrates (6).

The binding-induced change of the overall structure of PBPs might be the prerequisite for the adaptability and large coverage of different ligands by PBPs. The two conformations enable the positioning of ligands at the solvent-excluded and low dielectric protein interior (closed) while still allowing binding site residues to be placed at the evolving sites of protein surfaces (open) (4).

To gain insight into the evolution and designability of ligand binding and specificity, we analyzed the homologous PBPs, PotF and PotD, which are *Escherichia coli* putrescine (PUT)-binding protein and spermidine (SPD)-binding protein, respectively. They constitute the first elements of two separate multicomponent uptake systems (PotFGHI and PotABCD) to transport the cationic polyamines PUT and SPD across the cellular membrane (7, 8). PotF and PotD only share 35% overall sequence identity, but their binding pockets as well as their respective ligands encompass a high structural similarity. Still, the protein-binding modes differ: PotF shows affinity for PUT and SPD, whereas PotD exclusively binds SPD. Prior to this work, we grafted the seven differences in amino acids from PotD binding pocket onto PotF (9); this resulted in PotF_SPD, which will be referred to as PotF/D throughout this work to keep naming for mutants more concise. PotF/D solely binds SPD, whereas PUT affinity is abolished. Nonetheless, this variant did not show an affinity for SPD as high as PotD. A structural intriguing feature of PotF/D (Protein Data Bank [PDB]: 7OYZ) is the semiclosed conformation of the ligated crystal structure. We only observed PotF to adopt a similar semiclosed conformation when binding the large polyamine spermine (PDB: 6YEC). The size of this ligand disrupts two salt bridges flanking the binding pocket (D_39_–R_254_ and R_91_–E_184_), which were deemed important for tight binding and ligand affinity (10). Upon closer inspection of the seven mutations in PotF/Ds crystal structure, two exchanges (D39E and S87Y) were identified to influence the wildtype-like salt bridge formation of PotFs between the lobes.

In our previous study on PotF (10), we deduced that binding events in PBPs are not narrowed down to two fixed conformations and a final ligand pose but are more dynamic and must be analyzed considering the overall protein dynamic as well. The observation that SPD affinity was solely maintained and PotF/Ds crystal structure adopted a semiclosed state led us to the approaches presented in this work. We conducted a combinatorial test of the sequence space between PotF-binding and PotF/D-binding pockets by dividing the pocket into three parts (proximal, distal, and aromatic box) to determine disadvantageous and beneficial residue changes. In addition, we approached the effects of more global changes on affinity by a stepwise reintroduction and reestablishment of the salt bridges. For both approaches, we utilized isothermal titration calorimetry (ITC) and X-ray crystallography as methods of analysis. Furthermore, we illustrate how a promiscuous PBP can quickly adapt high specificity and discuss the impact this phenomenon could have on pathogens like multidrug-resistant gram-negative bacteria.

## Results

### Polyamine-binding pockets in PotF and PotD

**Figure 1:**
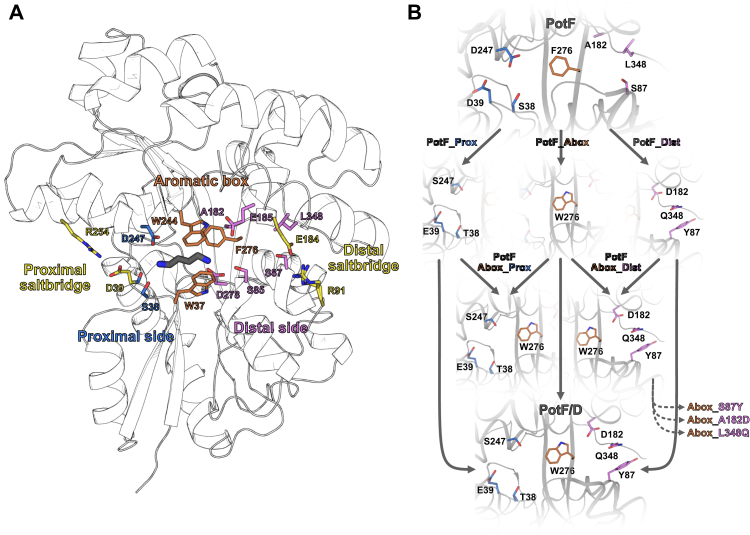
**PotF binding pocket and mutational steps towards PotF/D.***A*, binding pocket of PotF in complex with PUT (*gray sticks*). Proximal side chains are shown as *blue sticks*, aromatic box residues as *orange sticks*, and distal side chains as *pink sticks*. Salt bridges flanking the binding pocket are depicted as *yellow sticks*. Protein backbone is shown as *white cartoon with black outline*. *B*, schematic flowchart of the combinatorial sequence space analysis between PotF and PotF/D. In the first round, residues were grouped into proximal (PotF_Prox), aromatic box (PotF_Abox), and distal (PotF_Dist). Coloring of variants and residues was kept in relation with the figure. Binding pocket for PotF is illustrated from PotF in complex with PUT (Protein Data Bank ID: 6YE0), binding pocket of PotF/D and single groups is shown using PotF/D in complex with SPD (PDB ID: 7OYZ). Following the single-group analysis, proximal and distal residues were combined with the aromatic box substitution (PotF_Abox_Prox and PotF_Abox_Dist). Since the distal region seems to exert influence on ligand specificity, single mutants from the distal group in combination with the aromatic box were created and analyzed as well (PotF_Abox_S87Y, PotF_Abox_A182D, and PotF_Abox_L348Q). Protein structures were visualized using PyMOL (The PyMOL Molecular Graphics System; version 2.3; Schrödinger, LLC). PUT, putrescine; SPD, spermidine.

Despite great similarities among their binding pockets, PotF and PotD differ in their polyamine-binding profiles: PotF is promiscuous for PUT (*K*_*D*_ ≈ 68 nM) and SPD (*K*_*D*_ ≈ 30 μM) (10), whereas PotD binds exclusively SPD (*K*_*D*_ ≈ 6 nM) (9). We showed previously that this specificity can be swapped by exchanging respective residues in the binding pocket of PotF against their PotD counterparts (9). This resulted in PotF/D, which solely binds SPD (*K*_*D*_ ≈ 37 μM; Table 1). The mentioned PotF/D:SPD–*K*_*D*_ differs somewhat from our previously published one because of a changed setup (different ITC device and buffer conditions). Therefore, we remeasured all binding constants to keep comparability extremely high during the whole dataset of this study and to be able to interpret also slight differences in affinity and thermodynamics. Nonetheless, PotF/D does not reveal the specific interactions that direct the ligand-binding profiles of PotF and PotD for the different polyamines. To understand why this is the case, we analyzed the contributions to specificity from first shell mutations in the binding pocket using targeted mutagenesis and ITC. Comparison of the binding pockets of PotF and PotD suggests three distinct groups based on their location and ligand interaction (Table S1). Residues of the primary amine-binding site interacting with N1 of PUT and SPD in PotF were assigned to the proximal group (S38, D39, and D247; residues and numbering according to the PotF sequence without periplasmic signal peptide). Aromatic residues, which anchor the methylene backbone and N2 of the ligands *via* van der Waals, CH–π, and cation–π interactions, are assigned to the central aromatic box (W37, W244, and F276). Finally, residues forming direct interactions with N2 of PUT and N3 of SPD in PotF as well as equivalent residues forming interactions with N3 of SPD in PotD are assigned to the distal group (S85, S87, A182, E185, D278, and L348; Fig. 1). Aromatic box residues are conserved in both PBPs, the only difference is F276 in PotF that is substituted by tryptophan (W255) in PotD. Similarly, both proteins tightly coordinate the proximal primary amine of their ligands. The main differences between both proteins are found at the distal side. In PotD, polar residues (S83, Y85, D168, E171, and Q327) form ionic and hydrophilic contacts to the cationic N3 of SPD, whereas the distal binding pocket of PotF is slightly more hydrophobic in its characteristics (S85, S87, A182, E185, and L348) and shows a tightly coordinated water network (10). Three variants were generated based on the clustering of the active site: PotF_Prox (S38T, D39E, and D247S), PotF_Dist (S87Y, A182D, and L348Q), and PotF_Abox (F276W) (Fig. 1*B*).

**Table 1:** PUT and SPD affinities as determined by ITC for PotF and PotD as well as all variants constructed for the combinatorial mutation analysis between the sequences of PotF and PotF/D

Protein	Ligand	*K*_*D*_ (μM)	n	Δ*G* (kcal × mol^−1^)	Δ*H* (kcal × mol^−1^)	−*T*Δ*S* (kcal × mol^−1^)
PotF (10)	PUT	0.07 ± 0.04	0.90 ± 0.02	−9.74 ± 0.40	−23.02 ± 0.12	13.28 ± 0.52
	SPD	29.71 ± 1.15	0.92 ± 0.00	−6.07 ± 0.02	−3.65 ± 0.06	−2.42 ± 0.04
PotD (9)	PUT	N/D	—	—	—	—
	SPD	5.80 ± 1.2	0.86 ± 0.20	−11.13 ± 0.12	−16.40 ± 1.4	5.24 ± 1.39
PotF/D	PUT	N/D	—	—	—	—
	SPD	37.32 ± 2.4	0.94 ± 0.02	−5.94 ± 0.04	−9.30 ± 0.20	3.36 ± 0.24
PotF_Prox	PUT	N/D	—	—	—	—
	SPD	N/D	—	—	—	—
PotF_Abox	PUT	0.21 ± 0.04	0.90 ± 0.03	−8.97 ± 0.10	−22.25 ± 0.77	13.28 ± 0.75
	SPD	11.05 ± 0.88	0.96 ± 0.06	−6.65 ± 0.04	−6.25 ± 0.16	−0.40 ± 0.21
PotF_Dist	PUT	N/D				
	SPD	88.19 ± 33.10	0.91 ± 0.02	−5.48 ± 0.20	−1.83 ± 0.08	−3.65 ± 0.28
PotF_Abox_Prox	PUT	N/D	—	—	—	—
	SPD	N/D	—	—	—	—
PotF_Abox_Dist	PUT	N/D	—	—	—	—
	SPD	7.77 ± 0.32	0.94 ± 0.00	−6.85 ± 0.02	−5.28 ± 0.07	−1.57 ± 0.09
PotF_Abox-S87Y	PUT	14.92 ± 0.54	0.92 ± 0.03	−6.47 ± 0.02	−9.97 ± 0.20	3.50 ± 0.18
	SPD	19.95 ± 0.70	0.97 ± 0.02	−6.30 ± 0.02	−5.66 ± 0.12	−0.65 ± 0.12
PotF_Abox-A182D	PUT	83.22 ± 2.08	0.91 ± 0.01	−5.45 ± 0.02	−9.79 ± 0.09	4.33 ± 0.08
	SPD	3.13 ± 0.62	0.91 ± 0.02	−7.39 ± 0.11	−4.37 ± 0.20	−3.02 ± 0.15
PotF_Abox-L348Q	PUT	2.66 ± 0.08	0.94 ± 0.03	−7.48 ± 0.02	−14.22 ± 0.75	6.74 ± 0.74
	SPD	3.26 ± 0.14	0.98 ± 0.01	−7.83 ± 0.68	−7.72 ± 0.03	−0.11 ± 0.69

If the *K*_*D*_ values are not cited, all are measured in biological triplicates. The error is the standard deviation between the three measurements.

Abbreviation: N/D, not determinable.

### Residue influence on polyamine specificity in PotF mutants

PUT and SPD binding profiles of the generated variants were analyzed by ITC. The proximal and distal cluster mutagenesis (PotF_Prox and PotF_Dist) abolishes association of PUT, whereas only PotF_Dist maintained a very low SPD affinity (Table 1). The single mutation F276W in the aromatic box increases SPD binding about threefold (*K*_*D*_ ≈ 11 μM), whereas PUT affinity decreases threefold (*K*_*D*_ ≈ 0.21 μM) compared with wildtype PotF. The single mutation F276W does not result in exclusive SPD binding as it is observed for PotF/D (Table 1). The affinity-modulating effect of an equivalent position was also observed for the respective homologs of PotD and PotF, SpuD (PotF, sequence identity: 57.8%) and SpuE (PotD, sequence identity: 34%), in *Pseudomonas aeroguinosa* (11).

Because of the key role of F276W in affinity modulation, F276W was combined with the proximal and distal mutations (Fig. 1*B*), respectively. The PotF_Abox_Prox variant (S38T, D39E, D247S, and F276W) showed no binding of PUT similar to PotF_Prox but restored a very low SPD affinity (Table 1). This illustrates the power of F276W in relation to SPD binding and the importance of the proximal residues that form interactions with the N1 primary amines of the respective ligands for polyamine binding in general. This is in accordance with previous mutational studies (12, 13) as well as molecular dynamic simulations, where the proximal side was the first responding region upon ligand encounter (10). The combination of the distal substitutions (S87Y, A182D, and L348Q) with F276W in the aromatic box not only maintains SPD binding but also improves it almost twofold (*K*_*D*_(SPD) ≈ 7.8 μM) compared with PotF_Abox and roughly fourfold to fivefold compared with PotF and PotF/D, respectively. In addition, PUT affinity was not detectable. This proves that the distal residues in combination with F267W are the main switches to alter the specificity of PotF toward SPD. This is in line with the study of Machius *et al.* (14), who analyzed a PotD homolog from *Treponema pallidum* (TpPotD) and declared the architecture of the distal part of the binding pocket as one principle to explain polyamine specificity and in particular SPD preference. Conversely, the authors link PUT preference to a tighter anchoring of N1. However, this second principle seems questionable since SPD is also bound by wildtype PotF, and the exchanges of the proximal residues abolished the affinity for both polyamines.

We combined each single distal mutation (S87Y, A182D, or L348Q) with F276W (Abox, Fig. 1*B*) to evaluate the specific contributions of these residues further. All three variants show polyamine promiscuity, albeit with different apparent affinities (Table 1). The most prominent swap of the original preference for PUT over SPD and the highest affinity for SPD along this mutational approach is achieved through the combination of A182D with F276W. It exhibits a roughly tenfold increase in affinity for SPD (*K*_*D*_(SPD) ≈ 3.1 μM) with a concomitant decrease of PUT affinity by around 1200-fold (*K*_*D*_(PUT) ≈ 83 μM) compared with wildtype PotF. The variant based on L348Q shows the same tendency but less pronounced (*K*_*D*_(SPD) ≈ 2.6 μM, *K*_*D*_(PUT) ≈ 3.3 μM), whereas S87Y has a negative effect on both polyamines compared with the single mutation F276W. In summary, we can achieve high SPD affinity by only switching two amino acids in the binding pocket of PotF, of which F276W is important to maintain affinity in general and A182D or L348Q to improve affinity. For SPD specificity, the combinatorial synergy of multiple discriminatory mutations is important.

In addition to determining the apparent binding constants, ITC unravels the underlying thermodynamic contributions. We previously described highly conserved and coordinated water molecules in the PotF-binding pocket that are thermodynamically favorable (10, 15). They serve as placeholders for ligand molecules and can be displaced upon binding. Two water molecules are removed when accommodating the propylamine extension of SPD compared with PUT, which results in higher solvation entropy during the binding process (Fig. S1). In addition, hydrophobicity of the binding pocket increases, if more water molecules (solvent) are released, which is also linked to more entropically driven interactions (16). This is in line with all tested PotF-binding pocket mutants in which the thermodynamic properties for SPD remain enthalpically and entropically favored, albeit to varying degrees. For example, the gain of PotF_Abox in enthalpic shares (ΔΔ*H* = −2.65 kcal/mol) for binding SPD is accompanied by a large loss in the favorable entropic contribution (Δ(−*T*Δ*S*) = +2.02 kcal/mol) compared with the wildtype (Table 1), which could be explained through tighter stacking interactions of tryptophan with the longer ligand SPD. In contrast, PUT binding is exclusively driven by an enthalpic term that compensates for unfavorable entropies. In PotF_Abox, PUT binding solely shows a loss of enthalpy contributions (ΔΔ*H* = +0.77 kcal/mol) compared with the wildtype, whereas the unfavorable entropy term stays remarkably similar (Δ(−*T*Δ*S*) = ±0 kcal/mol; Table 1). Other interesting variants from a thermodynamic point of view are PotF_Abox_L348Q and PotF_Abox_S87Y. These constructs roughly display the same affinity for both polyamines and consequently a similar Gibbs free energy but show completely different thermodynamic profiles. This appears to be a textbook example for entropy–enthalpy compensation (17). The changes in enthalpy and entropy upon polyamine binding in PotF_Abox_L348Q or S87Y are too big compared with the neglectable change in Gibbs energy to be due to only conformational changes, which is the conventional explanation for entropy–enthalpy compensation. Hence, they must partially result from variations in the amounts of water immobilized or released upon complex formation (18). This underlines the importance of water molecules in the binding pocket of PotF and their role in polyamine recognition and specificity.

Taken together, the ITC measurements show that the polyamine specificity is predominantly encoded within the aromatic box residue 276 and the distal positions 182 and 348 of the PotF binding pocket. Furthermore, the distal residues seem to exert their influence on binding specificity *via* thermodynamically favoring or disfavoring the stabilization of water molecules instead of N3 of SPD.

### Approaching polyamine specificity from the conformational perspective of the proteins

**Figure 2:**
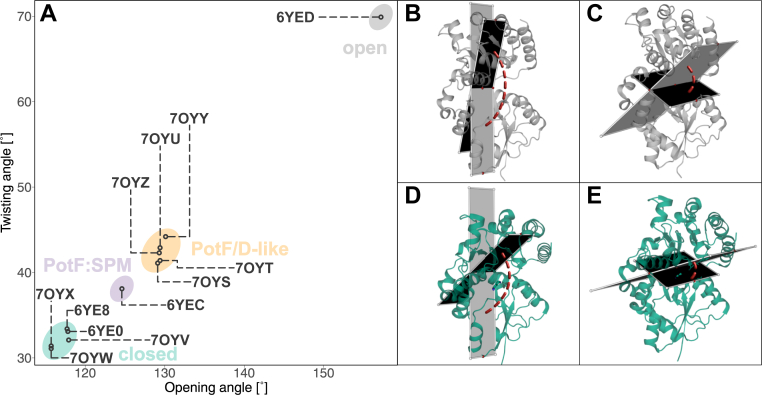
**Opening and twisting angles of crystallized variants.***A*, plot of opening and twisting angles of all variants, where we solved an X-ray structure. Each *circle* corresponds to the Protein Data Bank (PDB) identifier it is linked to. *B*–*E*, illustration of how the opening and twisting angles were determined. Opening angle for (*B*) PotF apo open (PDB ID: 6YED) and (*D*) PotF:PUT (PDB ID: 6YE0) and, respectively, twisting angle for PotF apo (*C*) and PotF:PUT (*E*). Planes in *gray* depict the dihedral angle between the Cα atoms of the four chosen residues. Twisting and opening angles were calculated according to Kröger *et al.* between residues 55, 136, 220, and 276 and 63, 276, 313, and 361, respectively. Protein structures were visualized using PyMOL (The PyMOL Molecular Graphics System, version 2.3; Schrödinger, LLC). PUT, putrescine.

Beyond the contribution of the individual positions in the binding pocket, the opening of the structure is essential; it influences all residues lining the binding pocket and the accessibility for mediating water molecules. The formation of salt bridges and the resulting stabilization of the closed state upon ligand binding seem to play a major role for ligand affinity in PotF, highlighting the influence of other important protein elements besides the binding pocket residues (10). PotF/D showed a more open conformation in the crystal structure (PDB: 7OYZ) when binding SPD (Fig. 2). A similar phenomenon, albeit less pronounced, was observed in PotF:spermine (PDB: 6YEC; Fig. 2*A*), in which interlobe salt bridges flanking the binding pocket D_39_–R_254_ and R_91_–E_184_ are disrupted by interactions with the ligand itself. Thereby, complete closure is hindered in these structures.

**Figure 3:**
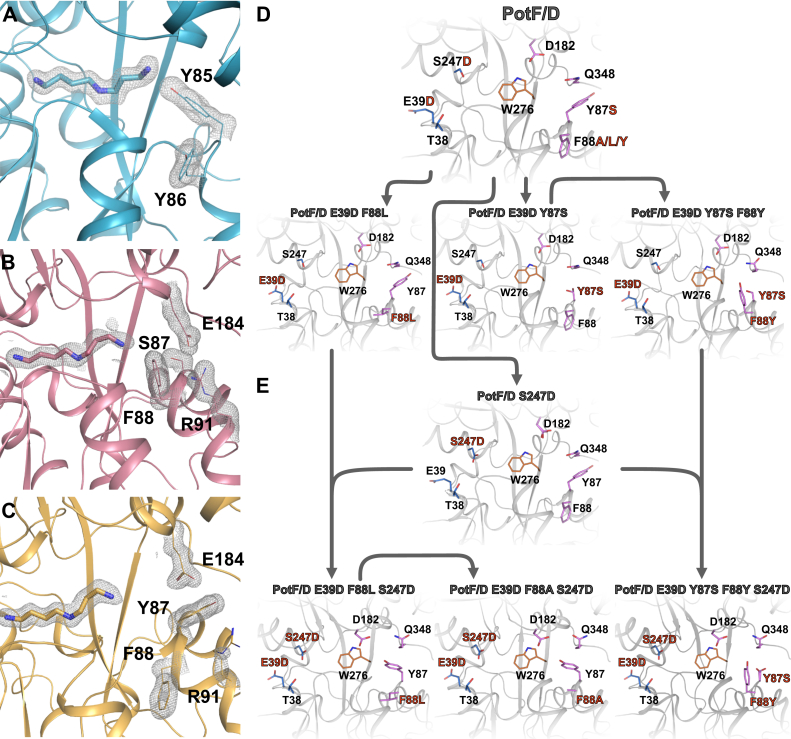
**Structural analysis of distal tyrosine and mutational steps on PotF/D.** Design puzzles of PotF/D and the interference for closure (*A*–*C*) as well as the design pathway for improvement (*D* and *E*). In PotD (*A*; Protein Data Bank [PDB] ID: 1POT), Y85 faces inward, directly coordinating SPDs N3. In wildtype PotF in complex with SPD (*B*; PDB ID: 6YE8), the corresponding position is occupied by S87 facing toward the binding pocket but not directly reaching SPD. F88 is in close proximity to S87. In the designed PotF/D (*C*; PDB ID: 7OYZ), the newly introduced Y87 to improve SPD binding faces away from the pocket and thereby disrupts the distal salt bridge (*E*_184_–R_91_). In addition, Y87 and F88 seem to repulse each other. *D*, shows the first steps of reintroducing wildtype-like salt bridge residues and changes made at position 88 to allow the initial design of Y87 to fit inside the pocket and allow for salt bridge formation in addition. The consecutive final design step following *D* to allow complete closure in our final constructs is shown in *E*. In *A*–*C*, SPD and important side chain residues are shown as sticks with their 2*F*_o_–*F*_c_ densities contoured at 1σ as *gray mesh*. Protein structures were visualized using PyMOL (The PyMOL Molecular Graphics System, version 2.3; Schrödinger, LLC). SPD, spermidine.

Closer examination of PotF/D revealed that two mutations interfere with wildtype-like salt bridge formation. Although D39E would increase the length of the one residue involved in the proximal salt bridge and partially open the structure, the exchange from Asp to Glu still allows salt bridge formation in general. However, the second mutation S87Y seemed to have a much bigger impact on the ligand recognition of PotF as shown in the prior analysis. In PotD, this Tyr faces inward into the binding pocket to coordinate N3 of SPD (Fig. 3*A*). In contrast, when introduced in PotF/D, this Tyr turns outward and disrupts the salt bridge between E184 and R91 (Fig. 3*C*). This observation explains the negative influence of S87Y on polyamine binding in general as well as the lack of affinity improvement in PotF/D, as the Tyr does not only disrupt the salt bridge and thereby obstructs complete closure but also is not able to coordinate N3 of SPD. Nevertheless, the unfavorable position of Y87 seemed irregular as, in principle, there should be space to accommodate this residue inside the binding pocket. This shifted our attention to the close surroundings of position Y87, in particular F88. In PotF (Fig. 3*B*), this Phe points inside the binding pocket, but in PotF/D, its rotamer is vertically flipped by ∼180°. It appears that F88 and Y87 repel each other in PotF/D, driving both residues away from the binding pocket and eliminating PotD-like positioning of Y87 (Fig. 3*C*). All prior observations combined led to the following three constructs: the basic double salt bridge reintroduction E39D–Y87S as well as E39D–F88A and E39D–Y87S–F88Y (Fig. 3*D*). The F88A containing variant was constructed to stay with the original plan of grafting the SPD-binding mode of PotD onto PotF. The removal of this Phe should allow Tyr to flip inside the binding pocket to accommodate PotD-like positioning. We also introduced F88Y in the basic double salt bridge mutant (E39D + Y87S), thereby hijacking a bulky aromatic residue that already points toward the binding pocket to introduce a distal PotD-like Tyr.

Upon analysis by ITC, an increased SPD affinity was observed for each of the constructed variants. A rudimentary but insignificant PUT affinity was observable in these variants compared with the original PotF/D and thereby still deemed as not determinable (Table 2) in our measurements. The F88A containing mutant showed a similar affinity for SPD as the double salt bridge construct but a different thermodynamic profile. Binding modes of constructs containing Y87S are again similar to the SPD binding of PotFs, revealing beneficial enthalpic and entropic shares (Table 2). On the other hand, the F88A carrying mutant shows a substantial increase in enthalpic and an unfavorable entropic contribution, in this case, more like PotF binding PUT (Table S2). The improvement of the enthalpic contribution by the F88A substitution could suggest a rearrangement of Y87 forming hydrophilic interactions or hydrogen bonds. The highest affinity was measured for the double salt bridge mutant containing the additional F88Y (*K*_*D*_(SPD) ≈ 5.3 μM). The introduction of F88Y changed the themodynamic profile of the double saltbridge construct toward the F88A variant albeit less pronounced (Table 2).

**Table 2:** PUT and SPD affinities as determined by ITC for PotF/D constructs

Protein	Ligand	*K*_*D*_ (μM)	n	Δ*G* (kcal × mol^−1^)	Δ*H* (kcal × mol^−1^)	−*T*Δ*S* (kcal × mol^−1^)
PotF/D–E39D–Y87S	PUT	N/D	—	—	—	—
	SPD	9.44 ± 0.91	0.98 ± 0.01	−6.74 ± 0.06	−6.56 ± 0.13	−0.18 ± 0.19
PotF/D–E39D–F88A	PUT	N/D	—	—	—	—
	SPD	9.60 ± 0.54	0.98 ± 0.03	−6.73 ± 0.03	−12.22 ± 0.35	5.49 ± 0.36
PotF/D–E39D–F88L	PUT	N/D	—	—	—	—
	SPD	9.90 ± 0.45	1.03 ± 0.01	−6.71 ± 0.03	−12.31 ± 0.11	5.60 ± 0.13
PotF/D–E39D–Y87S–F88Y	PUT	N/D	—	—	—	—
	SPD	5.32 ± 1.43	0.99 ± 0.02	−7.09 ± 0.15	−7.81 ± 0.21	0.71 ± 0.32
PotF/D–S247D	PUT	N/D	—	—	—	—
	SPD	9.43 ± 3.07	0.94 ± 0.01	−6.77 ± 0.18	−6.76 ± 0.08	−0.01 ± 0.25
PotF/D–E39D–F88A–S247D	PUT	125.90 ± 5.29	0.92 ± 0.04	−5.23 ± 0.02	−5.21 ± 0.14	−0.02 ± 0.15
	SPD	1.48 ± 0.14	0.91 ± 0.01	−7.82 ± 0.06	−4.75 ± 0.09	−3.07 ± 0.14
PotF/D–E39D–F88L–S247D	PUT	202.70 ± 43.13	0.90 ± 0.01	−4.97 ± 0.13	−5.54 ± 0.73	0.57 ± 0.86
	SPD	3.33 ± 0.53	0.93 ± 0.01	−7.35 ± 0.09	−4.19 ± 0.06	−3.17 ± 0.10
PotF/D–E39D–Y87S–F88Y–S247D	PUT	77.92 ± 3.17	0.90 ± 0.01	−5.51 ± 0.02	−4.15 ± 0.05	−1.36 ± 0.05
	SPD	0.84 ± 0.27	0.91 ± 0.02	−8.17 ± 0.17	−5.46 ± 0.08	−2.71 ± 0.16

*K*_*D*_ values are measured as biological triplicates. The reported error is the error between the three measurements.

Abbreviation: N/D, not determinable.

### Structural analysis of salt bridge constructs

In order to further investigate the mutant's contributions, we tried crystallization of the PotF/D constructs E39D–Y87S, E39D–Y87S–F88Y, and E39D–F88A. Unfortunately, we were not able to crystallize F88A after numerous attempts, while high-resolution structures (Table S3) for both other variants were acquired. It seemed the removal of a whole benzene ring in F88A and keeping only a methyl group destabilized the protein by creating a large unoccupied space. To counteract the aforementioned difficulties, we constructed a variant bearing F88L instead of F88A. This construct displays a slightly lower SPD affinity (*K*_*D*_(SPD) ≈ 9.9 μM; Table 2) while maintaining the general thermodynamic profile. It acts as a substitute to evaluate whether the structural positioning of Y87 can be altered by removing F88. We obtained high-resolution datasets for this variant in a similar fashion as for the other constructs (Table S3). To our surprise, all solved structures adopt a PotF/D-like semiclosed conformation (Fig. 2*A*).

**Figure 4:**
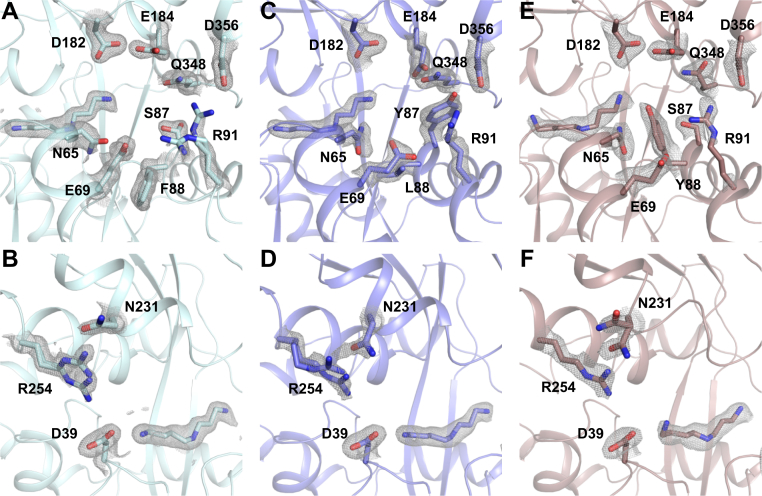
**Structural details of the first-round salt bridge mutants related to**Figure 3***D*.***A*, *C*, and *E*, distal side of the binding pocket of PotF/D–E39D–Y87S (*A*, Protein Data Bank [PDB] ID: 7OYS), PotF/D–E39D–F88L (*C*, PDB ID: 7OYT), and PotF/D–E39D–Y87S–F88Y (*E*, PDB ID: 7OYU). Proximal salt bridge side of the binding pocket of PotF/D–E39D–Y87S (*B*), PotF/D–E39D–F88L (*D*), and PotF/D–E39D–Y87S–F88Y (*F*). SPD and important side chain residues are shown as *sticks* with their 2*F*_o_–*F*_c_ densities contoured at 1σ as *gray mesh*. Protein structures were visualized using PyMOL (The PyMOL Molecular Graphics System, version 2.3; Schrödinger, LLC). SPD, spermidine.

Also, SPD adopts similar positions to the one in PotF/D in all structures. In E39D–Y87S, no specific differences to PotF/D are observable. Reverting S87 to Y did not result in complete closure or F88 flipping back inside the binding pocket (Fig. 4*A*). The same behavior is observed in the F88L variant where Leu tries to mimic Phe positioning (Fig. 4*C*). It seems that in these constructs, occupation of the space around residue 88 is important; this puts further emphasis on the problems regarding the F88A exchange. In F88L, Y87 remains flipped outside the distal region of the binding pocket. Tyr fills the unoccupied space between the salt bridge residues, interacts with R91 of the salt bridge, and stacks with Q348. Salt bridge residue E184 interacts with Q348 and Y87 as well (Fig. 4*C*).

Furthermore, R91 can interact with D356. In the E39D–Y87S structure (Fig. 4*A*), R91 shows an additional alternate conformation that can interact with E69. This interaction is possible in the completely open “apo” structure (PDB: 6YED) of wildtype PotF as well, hinting at an Arg “hand-off” between E69 or D356 and E184 upon closure.

At the proximal side, D39 is part of the water network involved in coordinating N1 of the ligand. The respective R254 can partially interact with D39 and N231. This time, R254 adopts an alternative conformation in E39D–Y87S and E39D–F88L, favoring one of the possible interactions in each (Fig. 4, *B* and *D*), respectively. The interaction with N231 supports the conformation of R254 in the semiclosed state of these structures and is observable in the open apo state of PotF wildtype again. This might hint at a second “hand-off” mechanism for salt bridge formation upon closure similar to the distal side.

In the highest affinity F88Y mutant, the newly introduced Tyr slightly rotates inside the binding pocket but does not directly interact with the ligand. It is involved in cation–π interactions with R91 and can stack with N65. The second salt bridge residue E184 interacts with D182 (Fig. 4*E*). Whether all these constructs can completely close in solution remains elusive. It is always possible that, with these specific mutations and the constraints they exercise, the semiclosed conformation is more favored for crystallization of these variants, since the main interplaying distal residues Y87, D182, and Q348, are all newly introduced in PotF/D. Nonetheless, more factors than just the two salt bridges need to influence the preference for complete closure.

### Joining the two approaches

To solve the conundrum of a completely closed PotF wildtype-like conformation for our designs, we looked at the dataset as a whole. The importance of the proximal side of the binding pocket and its role for polyamine binding in general stood out. We already addressed the minor influence of D39E and its reverse counterpart. The T38S exchange was deemed to have less of an influence since the general properties of the residue stayed constant with just an addition of a methyl group. Hence, the focus shifted toward D247S, the wildtype Asp bears one of two major carboxyl groups important in primary amine coordination upon ligand binding and recognition in PotF. In addition, there must still be a direct influence on closure at the proximal side even after reverting D39 to E.

We introduced the S247D exchange into PotF/D and into our high-affinity constructs E39D–Y87S–F88Y and E39D–F88A/L (Fig. 3*E*). PotF/D–S247D showed an improved SPD affinity in the regime of the first-round salt bridge construct (*K*_*D*_(SPD) ≈ 9.4 μM; Table 2). The combination of S247D with the other constructs led to an affinity improvement with which the nanomolar range was reached for the first time, while keeping the order of affinities in the different mutants the same compared with the prior analysis. All binding modes shifted toward being enthalpically and entropically favored, reflecting the wildtype binding mode of PotF for SPD (Table 2). Nonetheless, all constructs regained PUT affinity in the medium to low micromolar range (Table 2). This was expected as we reintroduced the wildtype-like proximal primary amine–binding site in these variants.

**Figure 5:**
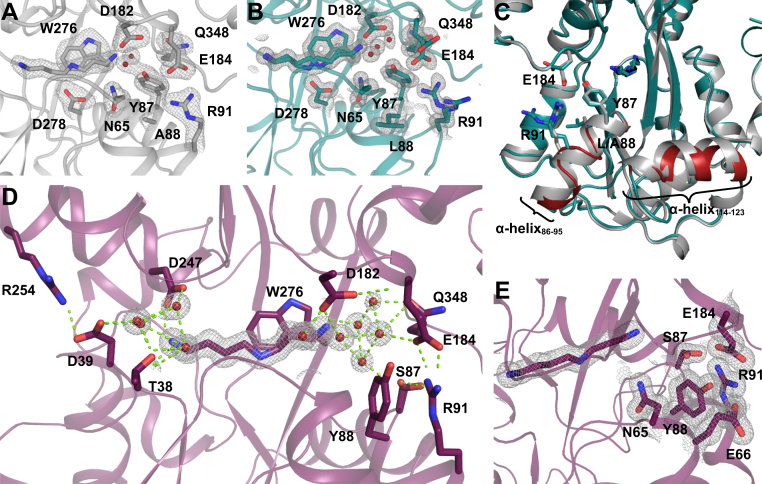
**Structural details of second-round salt bridge mutants related to**Figure 3***E*.***A*, PotF/D–E39D–F88A–S247D (Protein Data Bank [PDB] ID: 7OYV). *B*, PotF/D–E39D–F88L–S247D (PDB ID: 7OYW). *C*, PotF/D–E39D–F88L–S247D in *green* and PotF/D–E39D–F88A–S247D in *gray*. *Red* highlights the differences in the helices of PotF/D–E39D–F88L–S247D (*green*) to PotF/D–E39D–F88A–S247D (*gray*). *D*, PotF/D–E39D–Y87S–F88Y–S247D (PDB ID: 7OYX) with waters in the binding pocket shown as *red spheres* and the polar contacts highlighted as *light green dashed lines*. *E*, distal side of the binding pocket of PotF/D–E39D–Y87S–F88Y–S247D highlighting the tight and pocket-like coordination of Tyr88. SPD and important side chain residues are shown as *sticks*. If densities are shown, 2*F*_o_–*F*_c_ maps contoured at 1σ are depicted as *gray mesh*. Protein structures were visualized using PyMOL (The PyMOL Molecular Graphics System, version 2.3; Schrödinger, LLC). SPD, spermidine.

We successfully obtained X-ray data for all newly generated variants including the F88A carrying construct (Table S3). All structures but PotF/D–S247D adopt a fully closed wildtype conformation (Fig. 2). This backs up the theory that major elements on either side of the binding pocket play a crucial role in modulating closure, excluding the proximal salt bridge since it never truly was disrupted but rather extended. In the F88L/A variants, Y87 is finally able to rotate toward the binding pocket and allows distal salt bridge formation. The residue, however, does not coordinate the primary amine at N3 of SPD directly but takes part in a hydrogen bonding network with D182, N65, and a water molecule, which is a direct interaction partner to N3 of SPD (Fig. 5, *A* and *B*).

The orientation of the residues in both mutants is identical, confirming the F88L containing variant as a structural example for the noncrystallizable F88A construct in the first part of this study. An interesting feature of the F88A containing construct is the orientation of the N2 of SPD, as it faces toward the aromatic box residues to form stacking interaction in contrast to all other constructs, in which interaction with D278 is always preferred. The F88A containing structure is less resolved than the others, and the ligand density could allow for both conformations of N2 in the density, but multiple refinement cycles ended up preferring the built one. This puts more emphasis on the remaining question regarding the slightly lower affinity of F88L. As previously described, Leu at position 88 rotates downward, similar to Phe in the original PotF/D, thereby α-helix 86 to 95 slides back and loses typical α-helical properties as determined by DSSP (Fig. 5*C*; (19)). This slight structural change is propagated over two loops and α-helix 114 to 123 with an RMSD (over all Cαs) of 0.610 Å for this specific region compared with the residues before (29–86, 0.272 Å), after (124–369, 0.335 Å), as well as the complete chain A RMSD of 0.481 Å. Chain B as well as the A–B chain comparison behaves in a comparable manner (Table S2). This deviation puts extra strain on the structure of F88L and especially the distal salt bridge since R91 is in the affected region. In general, the noise in the *F*_o_–*F*_c_ map of this structure seems to indicate many flexible side chains. The “flexible” F88L structure is also the first in which we ever observed SPD taking alternate conformations in the binding pocket of which one does not mimic PUT-like positioning. PotF/D–E39D–Y87S–F88Y–S247D is the construct with the highest SPD affinity (840 nM; Fig. 5*D* and Table 2). In this structure, Y88 is stacking with N65 and the salt bridge residue R91, stabilizing the latter and thereby adopting the fully closed conformation. Furthermore, the hydroxyl group of Y88 is coordinated by E66, whereas R91 is interacting with S87 in two alternate conformations (Fig. 5*E*). Taken together, the interplay of the mentioned residues forms a tight pocket surrounding Y88, thereby stabilizing the distal region and supporting the completely closed conformation. In all constructs that carry S247D, SPD adopts a “relaxed” conformation and does not show unexpected bending of its backbone to fit a specific position as it does when being bound by PotF wildtype or PotF/D (Fig. 5, *A*, *B*, and *D*).

All in all, we determined salt bridges and the key carboxyl-harboring proximal residue D247 as the basic prerequisites for a complete closure of PotF constructs. Fixing each component by itself was not sufficient. Only the combination of both allows full closure, deeming it an intricately regulated process.

## Discussion

### Design aspects and interpretation

We were able to pinpoint SPD specificity to the aromatic box and the interplay of several distal residues. Nonetheless, higher affinity SPD binders always came at a partial loss of specificity by reintroducing a slight PUT affinity. In most cases, PUT affinity remained marginal and at best in high micromolar ranges. This affinity is detectable in *in vitro* measurements, whereas in an *in vivo* situation our final variants would almost always prefer SPD binding over PUT if given the choice. On the one hand, we were able to develop an improved specific SPD-binding PotF variant in PotF_Abox_Dist (7.8 μM; Table 1) as well as a whole variety of enhanced PotF/D constructs (E39D–Y87S, E39D–F88A, E39D–F88L, E39D–Y87S–F88Y, and S247D) with good SPD affinity (9.4, 9.6, 9.9, 5.3, & 9.4 μM, respectively) and an array of different thermodynamic binding profiles (Table 2). On the other hand, we designed a PotF/D construct that completely switched its ligand preference compared with the wildtype by exhibiting nanomolar affinity for SPD (840 nM) and medium micromolar affinity for PUT (88 μM). It appears that shaping affinity comes always at the risk of losing specificity and vice versa.

### Implications for evolutionary adaptation of polyamine uptake in pathogenic bacteria

Promiscuity of a receptor allows recognition of a range of structurally and chemically similar molecules; this comes with the advantage of adaptability for the host organism as well, hence it is less susceptible to changes exerting selective pressure. Nonetheless, this adaptability bears negative aspects, especially looking at pathogens. In the case of PotF and PotD, numerous orthologs with good sequence conservation, especially in the binding pocket, have been identified. Highly similar receptors have been characterized for polyamine specificity in different bacterial species (14, 20). PotD has been identified as a potential virulence factor in *Streptococcus pneumoniae* (21) and successfully applied as an immunization against systemic infection in mice (22). Multidrug-resistant gram-negative bacteria have a significant impact on public health, and SPD uptake has been linked to the expression of type III secretion (T3SS) system genes (23), which are an essential part in their pathogenesis (24, 25). Therefore, a SpuE antibody was designed to prevent SPD transport and ultimately weakening *Pseudomonas aeruginosa* infection (26). Bacterial pathogens have proven their adaptability numerous times, and by having promiscuous PBPs like PotF (or its homologs) at their disposal, they might just be able to hijack another system to facilitate SPD uptake and bypass treatment methods in the long run. Two mutations in PotF_Abox_A182D are enough to reverse polyamine specificity in PotF to favor SPD. This does not take into consideration the already mediocre micromolar (30 μM) affinity of PotF for SPD, which could explain why a previous study on a *ΔpotD S. pneumoniae* strain (21) already hinted at the existence of an alternative SPD uptake system. It is conceivable that this might have been the co-usage of the PotFGHI system by both polyamines. This is not uncommon in nature, since two other highly similar PBPs (70% sequence identity (27)), LAOBP and HisJ, both of which show promiscuous binding capabilities for Lys, Arg, Orn, and His but in a different order (28, 29, 30), even share the same inner membrane ATPase-permease complex (HisQMP_2_; (27)). Major findings of our study compared with others on the PBPs of the polyamine uptake system are in mutual agreement, thus it can be feasible to extrapolate these results onto other bacterial species in which homologs or orthologs of these proteins can be found. This might suggest looking at PotD and PotF as well as their respective relatives in more of a joint manner from a medical perspective.

### Conservation and evolution of polyamine transporter systems

**Figure 6:**
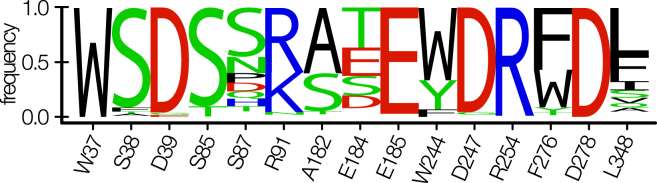
**WebLogo representation of the conservation analysis of PotF homologs**.

To put further emphasis on this point, we utilized a protein BLAST on the UniProt entry of PotF (UniProt ID: P31133), which resulted in 250 unbiased hits of polyamine transporter systems from numerous different organisms (Material S1). We analyzed conservation and mutational frequency of important binding pocket and salt bridge residues. This resulted in a median conservation of ∼75% for all 15 residues. The highest conservation is present for aromatic box residue W37 (100%) and key carboxyl harboring residues D247, D278, and E185 as well as proximal salt bridge residues D39 and R254 (all ≥96%). Interestingly, in a third of the sequences, F276W is present, and with generally less conservation at the distal side of the binding pocket (S87: 45%, A182: 60%, and L348: 43%), an evolution toward the aforementioned co-usage of PotF by multiple polyamines is conceivable. This is further supported considering PotF sequences being annotated as PUT/SPD transporter systems for several of the different organisms. The lowest conservation percentage shows distal salt bridge residue E184 with 26%. In 10% of the cases, E184D would still allow for salt bridge formation, but E184T (37%) seems to be the preferred evolutionary trajectory for this residue. Nonetheless, the opposing salt bridge residue R91 shows good conservation with 59% and R91K being the nearest possible exchange with 37%, therefore keeping the possibility of distal salt bridge formation (E/D_184_–R/K_91_) in over a third of the cases. The results of the conservation analysis are visualized in Figure 6 as a WebLogo.

The lower conservation rate throughout many of the distal residues and the potential loss of salt bridge formation enable the distal part of the binding pocket to be less structurally fixed and, thus, allows for more flexibility and thereby adaptability for a broader ligand spectrum. We know already that PotF can recognize multiple polyamines on top of PUT and SPD (10), and the aforementioned findings suggest the same for different orthologs, which might have evolved toward even increased promiscuity by being exposed to various external stimuli throughout evolution.

### Further medical relevance

Polyamines are not only important for bacterial regulatory pathways but are involved in a multitude of processes in eukaryotes (31, 32, 33). Their presence and metabolism are commonly upregulated and dysregulated in cancer cells (34, 35). Therefore, polyamines have become the target of multiple treatment strategies as well as biomarkers for tumor progression in specific cancer types (34). In recent years, the usage of PBPs as receptor modules fused to circularly permuted fluorescent proteins as biosensors became a well-established approach to track small molecules (36, 37, 38). Our studies on PotF provide the perfect framework for engineering similar biosensors with desired affinities and specificities to assist clinical polyamine research.

## Experimental procedures

### Cloning of PotF and PotF/D variants

The gene for PotF (UniProt ID: P31133) without the N-terminal signal peptide was amplified by PCR using genomic DNA of *E. coli* K-12 and primers, which introduced flanking restriction sites for NdeI and XhoI. After digestion, the DNA fragment was ligated into a pET21b(+)-vector, thereby adding a C-terminal His6-tag. PotF/D (previously called PotF_SPD) was constructed prior to this study (9). Mutations for the construction of different PotF and PotF/D variants were introduced by a modified QuickChange PCR utilizing KAPA polymerase (Roche) followed by an additional ligation step using T4 DNA ligase (New England Biolabs) according to the manufacturer's protocol. All used oligonucleotides can be found in Table S4. Top10 cells were transformed with reaction mixture *via* heat shock and plated on LB agar containing ampicillin (100 μg/ml) as selection marker. Overnight cultures were grown in LB medium containing ampicillin (100 μg/ml), and DNA was isolated using NucleoSpin Plasmid EasyPure-Kit (Machery & Nagel) according to the manufacturer's protocol. To confirm correct implementation of mutations, plasmids were sequenced (Eurofins Genomics) using standard T7 primers. For expression of proteins, BL21 (DE3) cells were transformed with the plasmid and plated out on LB agar plates containing 100 μg/ml ampicillin as selection marker to obtain single colonies, which were further used to inoculate overnight cultures.

### Protein expression

For protein expression, 2 l LB were inoculated with 20 ml of an overnight culture containing 100 μg/ml ampicillin and incubated at 37 °C until an absorbance reached a value of 0.6 to 0.8 at 600 nm. Overexpression was induced by adding IPTG to a final concentration of 1 mM and further incubation for 4 h at 37 °C. Cells were harvested by centrifugation (Beckman Coulter; JLA 8.1000; 4000*g*, 20 min, 4 °C), and pellets were resuspended and washed with 30 ml buffer (50 mM Tris [pH 8], 300 mM NaCl, and 20 mM Imidazole). After recentrifugation (Eppendorf 5920R; S-4x750; 4000*g*, 1 h, 4 °C), the washed pellets were stored at −20 °C until further use.

### Protein purification

Cells were resuspended (50 mM Tris [pH 8], 300 mM NaCl, and 20 mM imidazole) and lysed by sonication (Branson; 6.3 mm tip, 3 × 3 min, 40% duty cycle, output power 4), followed by a centrifugation step (Beckman Coulter; JA25.50; 40,000*g*, 60 min) to separate the soluble from the insoluble fraction and cell debris. The supernatant was loaded onto an equilibrated (50 mM Tris [pH 8], 300 mM NaCl, and 20 mM imidazole) HisTrap HP 5 ml column (GE Healthcare) using a peristaltic pump (Pump P-1; GE Healthcare). After washing for ten column volumes with lysis buffer, unfolding of the protein to wash off endogenous ligands was achieved by washing and incubating for an hour with 6 M guanidinium hydrochloride and subsequently washing with ten column volumes of lysis buffer for refolding. The protein was eluted by a stepwise increase of the imidazole concentration to 260 mM with an ÄKTA system. Fractions containing the protein were pooled and concentrated with a centrifugal concentrator (Amicon; 10 kDa molecular weight cutoff) to a maximum volume of 12 ml and applied to an equilibrated (50 mM Tris [pH 8] and 300 mM NaCl) preparative size exclusion column (HiLoad Superdex 75 26/60; GE Healthcare). Fractions with monomeric protein were pooled and concentrated for ITC measurements or crystallization setups (Amicon; 10 kDa molecular weight cutoff). Protein concentration was determined photometrically using the absorption at 280 nm. Expression and purification were verified by SDS-PAGE.

### ITC

Ligands were always freshly prepared in the exact same buffer as was used for the size exclusion run. Samples were degassed, and temperature was equilibrated using a degassing station (TA Instruments). About 400 μl protein sample of different concentrations (Table S5) depending on affinity was transferred into the sample cell of a nanoITC (TA Instruments), and 50 μl of about tenfold concentrated ligand solution was loaded into the injection needle. Multiple injection measurements were carried out at 293 K, 300 rpm stirring rate, and 250 s spacings between each 2 μl injection. The heat quantity past injection was determined by integration of the measured peaks. Every protein–ligand combination was measured as biological triplicate. Subtraction of heat of dilution measurements, peak integration, and one site binding fits were done with NanoAnalyze (TA Instruments). Reported errors are the errors between fits of the measured triplicates.

### Crystallography

All crystallization experiments were set up as sitting drop vapor diffusion experiments in either 3-well Intelliplates (Art Robbins Instruments) or MRC Maxi plates (Swissci) using a protein concentration of 40, 30, or 15 mg/ml and a 20-fold molar excess of ligand. Protein–ligand mixtures were equilibrated at 293 K for several hours before crystallization setups.

First crystals were obtained after an initial sparse matrix screen using the commercially available JCSG CORE I-IV screens (QIAGEN). If needed, these hits were further optimized by using a grid screen to improve promising conditions and applying the Additive Screen (Hampton Research).

This resulted in conditions containing 2.4 M ammonium sulfate, 0.1 M bicine, pH 8.3 or 9.0, and either 4.5% or 5% Jeffamine M-600 as additive. We also obtained crystals in 0.1 M sodium acetate (pH 4.6), 0.2 M ammonium acetate, and 30% PEG 4000; 0.085 M sodium acetate [pH 4.7], 0.17 M ammonium acetate with 30% PEG 4000 and 15% glycerol; and 0.1 M MES (pH 5) with 30% PEG 6000. Detailed information regarding each crystal can be found in Table S6.

Crystals were mounted using CryoLoops and transferred into a cryogenic solution made of reservoir solution and either 25% glycerol or 1.7 M malonate matching the pH of the condition (Table S6) and then cooled down in liquid nitrogen. Data collection at 100 K was done at the beamlines BL 14.1 and 14.2 at the synchrotron BESSY II, Helmholtz-Zentrum Berlin (39). Diffraction data were processed using XDSAPP (40), and decision of resolution cutoff was made according to CC_1/2_ around 0.25, I/Sigma >0.5, and completeness in the outer shell >75%. Molecular replacement was done using Phaser-MR (14) with both lobes independently and missing hinge region residues of PotF (PDB: 1A99 (13)) as search model, to account for different opening angles. Data quality was assessed utilizing phenix.xtriage. Refinements were done with Phenix.refine (41). Manual model building was performed in Coot (42). Final models were evaluated by PDB_REDO 6.00 (43). Refinement statistics and crystallographic data are shown in Table S3.

### Bioinformatics analysis of PotF and related sequences

BLAST was used on the UniProt ((44); 2021) entry of PotF (POTF_ECOLI; P31133) with the settings listed in Table 3. The resulting hits were analyzed on the mutational distribution of each residue of interest for this study. A detailed list of all analyzed sequences and their respective organisms as well as their score, identity, and E-value with regard to PotF can be found in Material S1.

**Table 3:** pBLAST parameters used for the conservational analysis of PotF and PotF-related structures

Program	BLASTP (BLASTP 2.9.0+)
Database	uniprotkb_refprotswissprot (Protein) generated for BLAST on December 2, 2020
Sequences	57,391,823 sequences consisting of 21,838,191,652 letters
Matrix	blosum62
Threshold	10
Filtered	False
Gapped	True
Maximum no. of hits reported	250

## Data availability

X-ray coordinates of all solved structures have been deposited at the PDB (http://www.rcsb.org) with accession codes: 7OYS, 7OYT, 7OYU, 7OYV, 7OYW, 7OYX, 7OYY, and 7OYZ.

## Supporting information

This article contains supporting information.

## Conflict of interest

The authors declare that they have no conflicts of interest with the contents of this article.

## References

[1] Nobeli I., Favia A.D., Thornton J.M. (2009). Protein promiscuity and its implications for biotechnology. Nat. Biotechnol..

[2] Schreiber G., Keating A.E. (2011). Protein binding specificity versus promiscuity. Curr. Opin. Struct. Biol..

[3] Borrok M.J., Zhu Y., Forest K.T., Kiessling L.L. (2009). Structure-based design of a periplasmic binding protein antagonist that prevents domain closure. ACS Chem. Biol..

[4] Dwyer M.A., Hellinga H.W. (2004). Periplasmic binding proteins: A versatile superfamily for protein engineering. Curr. Opin. Struct. Biol..

[5] Felder C.B., Graul R.C., Lee A.Y., Merkle H.-P., Sadee W. (1999). The venus flytrap of periplasmic binding proteins: An ancient protein module present in multiple drug receptors. AAPS PharmSci..

[6] Moussatova A., Kandt C., O'Mara M.L., Tieleman D.P. (2008). ATP-binding cassette transporters in Escherichia coli. Biochim. Biophys. Acta.

[7] Furuchi T., Kashiwagi K., Kobayashi H., Igarashi K. (1991). Characteristics of the gene for a spermidine and putrescine transport system that maps at 15 min on the Escherichia coli chromosome. J. Biol. Chem..

[8] Pistocchi R., Kashiwagi K., Miyamoto S., Nukui E., Sadakata Y., Kobayashi H., Igarashi K. (1993). Characteristics of the operon for a putrescine transport system that maps at 19 minutes on the Escherichia coli chromosome. J. Biol. Chem..

[9] Scheib U., Shanmugaratnam S., Farías-Rico J.A., Höcker B. (2014). Change in protein-ligand specificity through binding pocket grafting. J. Struct. Biol..

[10] Kröger P., Shanmugaratnam S., Ferruz N., Schweimer K., Höcker B. (2021). A comprehensive binding study illustrates ligand recognition in the periplasmic binding protein PotF. Structure.

[11] Wu D., Lim S.C., Dong Y., Wu J., Tao F., Zhou L., Zhang L.-H., Song H. (2012). Structural basis of substrate binding specificity revealed by the crystal structures of polyamine receptors SpuD and SpuE from Pseudomonas aeruginosa. J. Mol. Biol..

[12] Kashiwagi K., Pistocchi R., Shibuya S., Sugiyama S., Morikawa K., Igarashi K. (1996). Spermidine-preferential uptake system in Escherichia coli. Identification of amino acids involved in polyamine binding in PotD protein. J. Biol. Chem..

[13] Vassylyev D.G., Tomitori H., Kashiwagi K., Morikawa K., Igarashi K. (1998). Crystal structure and mutational analysis of the Escherichia coli putrescine receptor. Structural basis for substrate specificity. J. Biol. Chem..

[14] Machius M., Brautigam C.A., Tomchick D.R., Ward P., Otwinowski Z., Blevins J.S., Deka R.K., Norgard M.V. (2007). Structural and biochemical basis for polyamine binding to the Tp0655 lipoprotein of Treponema pallidum: Putative role for Tp0655 (TpPotD) as a polyamine receptor. J. Mol. Biol..

[15] Schiebel J., Gaspari R., Wulsdorf T., Ngo K., Sohn C., Schrader T.E., Cavalli A., Ostermann A., Heine A., Klebe G. (2018). Intriguing role of water in protein-ligand binding studied by neutron crystallography on trypsin complexes. Nat. Commun..

[16] Du X., Li Y., Xia Y.-L., Ai S.-M., Liang J., Sang P., Ji X.-L., Liu S.-Q. (2016). Insights into protein-ligand interactions: Mechanisms, models, and methods. Int. J. Mol. Sci..

[17] Chodera J.D., Mobley D.L. (2013). Entropy-enthalpy compensation: Role and ramifications in biomolecular ligand recognition and design. Annu. Rev. Biophys..

[18] Dragan A.I., Read C.M., Crane-Robinson C. (2017). Enthalpy-entropy compensation: The role of solvation. Eur. Biophys. J..

[19] Kabsch W., Sander C. (1983). Dictionary of protein secondary structure: Pattern recognition of hydrogen-bonded and geometrical features. Biopolymers.

[20] Brandt A.-M., Raksajit W., Yodsang P., Mulo P., Incharoensakdi A., Salminen T.A., Mäenpää P. (2010). Characterization of the substrate-binding PotD subunit in Synechocystis sp. strain PCC 6803. Arch. Microbiol..

[21] Ware D., Jiang Y., Lin W., Swiatlo E. (2006). Involvement of potD in Streptococcus pneumoniae polyamine transport and pathogenesis. Infect. Immun..

[22] Shah P., Swiatlo E. (2006). Immunization with polyamine transport protein PotD protects mice against systemic infection with Streptococcus pneumoniae. Infect. Immun..

[23] Zhou L., Wang J., Zhang L.-H. (2007). Modulation of bacterial type III secretion system by a spermidine transporter dependent signaling pathway. PLoS One.

[24] Felise H.B., Nguyen H.V., Pfuetzner R.A., Barry K.C., Jackson S.R., Blanc M.-P., Bronstein P.A., Kline T., Miller S.I. (2008). An inhibitor of gram-negative bacterial virulence protein secretion. Cell Host Microbe.

[25] Keyser P., Elofsson M., Rosell S., Wolf-Watz H. (2008). Virulence blockers as alternatives to antibiotics: Type III secretion inhibitors against gram-negative bacteria. J. Intern. Med..

[26] Zhang Y., Sun X., Qian Y., Yi H., Song K., Zhu H., Zonta F., Chen W., Ji Q., Miersch S., Sidhu S.S., Wu D. (2019). A potent anti-SpuE antibody allosterically inhibits type III secretion system and attenuates virulence of Pseudomonas aeruginosa. J. Mol. Biol..

[27] Higgins C.F., Ames G.F. (1981). Two periplasmic transport proteins which interact with a common membrane receptor show extensive homology: Complete nucleotide sequences. Proc. Natl. Acad. Sci. U. S. A..

[28] Nikaido K., Ames G.F. (1992). Purification and characterization of the periplasmic lysine-, arginine-, ornithine-binding protein (LAO) from Salmonella typhimurium. J. Biol. Chem..

[29] Paul S., Banerjee S., Vogel H.J. (2017). Ligand binding specificity of the Escherichia coli periplasmic histidine binding protein, HisJ. Protein Sci..

[30] Pulido N.O., Silva D.-A., Tellez L.A., Pérez-Hernández G., García-Hernández E., Sosa-Peinado A., Fernández-Velasco D.A. (2015). On the molecular basis of the high affinity binding of basic amino acids to LAOBP, a periplasmic binding protein from Salmonella typhimurium. J. Mol. Recognit..

[31] Igarashi K., Kashiwagi K. (2010). Modulation of cellular function by polyamines. Int. J. Biochem. Cell Biol..

[32] Mandal S., Mandal A., Johansson H.E., Orjalo A.V., Park M.H. (2013). Depletion of cellular polyamines, spermidine and spermine, causes a total arrest in translation and growth in mammalian cells. Proc. Natl. Acad. Sci. U. S. A..

[33] Pegg A.E., Casero R.A. (2011). Current status of the polyamine research field. Methods Mol. Biol..

[34] Casero R.A., Murray Stewart T., Pegg A.E. (2018). Polyamine metabolism and cancer: Treatments, challenges and opportunities. Nat. Rev. Cancer.

[35] Soda K. (2011). The mechanisms by which polyamines accelerate tumor spread. J. Exp. Clin. Cancer Res..

[36] Marvin J.S., Borghuis B.G., Tian L., Cichon J., Harnett M.T., Akerboom J., Gordus A., Renninger S.L., Chen T.-W., Bargmann C.I., Orger M.B., Schreiter E.R., Demb J.B., Gan W.B., Hires S.A. (2013). An optimized fluorescent probe for visualizing glutamate neurotransmission. Nat. Methods.

[37] Marvin J.S., Schreiter E.R., Echevarría I.M., Looger L.L. (2011). A genetically encoded, high-signal-to-noise maltose sensor. Proteins.

[38] Marvin J.S., Shimoda Y., Magloire V., Leite M., Kawashima T., Jensen T.P., Kolb I., Knott E.L., Novak O., Podgorski K., Leidenheimer N.J., Rusakov D.A., Ahrens M.B., Kullmann D.M., Looger L.L. (2019). A genetically encoded fluorescent sensor for in vivo imaging of GABA. Nat. Methods.

[39] Mueller U., Förster R., Hellmig M., Huschmann F.U., Kastner A., Malecki P., Pühringer S., Röwer M., Sparta K., Steffien M., Ühlein M., Wilk P., Weiss M.S. (2015). The macromolecular crystallography beamlines at BESSY II of the Helmholtz-Zentrum Berlin: Current status and perspectives. Eur. Phys. J. Plus.

[40] Sparta K.M., Krug M., Heinemann U., Mueller U., Weiss M.S. (2016). XDSAPP2.0. J. Appl. Crystallogr..

[41] Adams P.D., Afonine P.V., Bunkóczi G., Chen V.B., Davis I.W., Echols N., Headd J.J., Hung L.-W., Kapral G.J., Grosse-Kunstleve R.W., McCoy A.J., Moriarty N.W., Oeffner R., Read R.J., Richardson D.C. (2010). PHENIX: A comprehensive Python-based system for macromolecular structure solution. Acta Crystallogr. D Biol. Crystallogr..

[42] Emsley P., Lohkamp B., Scott W.G., Cowtan K. (2010). Features and development of Coot. Acta Crystallogr. D Biol. Crystallogr..

[43] Joosten R.P., Joosten K., Murshudov G.N., Perrakis A. (2012). PDB_REDO: Constructive validation, more than just looking for errors. Acta Crystallogr. D Biol. Crystallogr..

[44] UniProt Consortium (2021). UniProt: The universal protein knowledgebase in 2021. Nucleic Acids Res..

