# Supplementary material for: Fine-tuning spermidine binding modes in the putrescine binding protein PotF
Source: J Biol Chem. 2021 Nov 19;297(6):101419. doi: 10.1016/j.jbc.2021.101419 (PMC8666671; doi:10.1016/j.jbc.2021.101419)
Supplement: Supplemental Figures S1–S3 and Tables S1–S6 [file mmc1.pdf]

Supplementary information for

Fine-tuning spermidine binding modes in the putrescine binding protein PotF

Pascal Kröger[1], Sooruban Shanmugaratnam[1,2], Ulrike Scheib[2], Birte Höcker[1,2*]

[1]Department for Biochemistry, University of Bayreuth, Bayreuth, Germany

[2] Max-Planck-Institute for Developmental Biology, Tübingen, Germany

*Corresponding author

Table of Content

Figure S1: Binding pocket of PotFwt:PUT and PotFwt:SPD with waters

Figure S2a: ITC measurements and analysis related to Table 1.

Figure S2b: ITC measurements and analysis related to Table 1.

Figure S3: ITC measurements and analysis related to Table 2.

Table S1: Residue groups in PotF and their respective counterparts in PotD

Table S2: RMS calculated by aligning the stated residues from PotF/D-E39D-F88A-S247D onto PotF/D-E39D-F88L-S247

Table S3: Crystallographic data and refinement statistics for all solved crystal structures

Table S4: Oligonucleotides used for the generation of PotF and PotF/D variants

Table S5: Concentrations of protein and ligand solutions used for the triplicate ITC measurements

Table S6: Full crystallization conditions, protein concentrations and cryogenic solutions used for the structure determination of all constructs

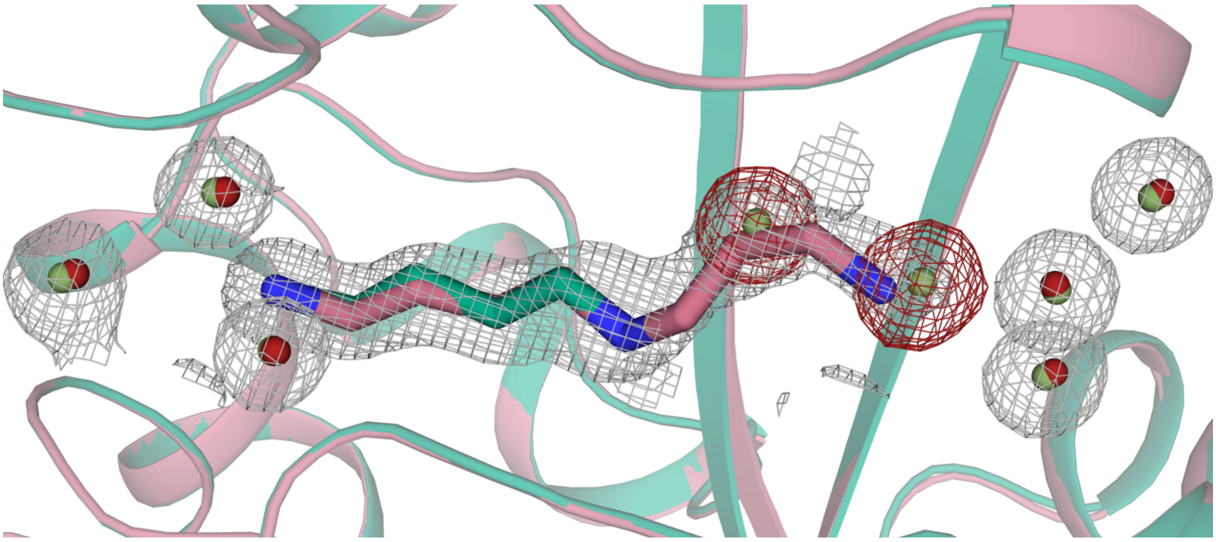

Figure S1: Binding pocket of PotFwt:PUT (green) and PotFwt:SPD (salmon) with waters (green and red spheres, respectively). Ligand molecules are depicted as sticks in the matching color. Maps for SPD and waters are shown as 2Fo-Fc-densities contoured at 1σ as gray mesh. The waters highlighted with a red mesh are displaced by SPD in PotFwt:SPD. Protein structures were visualized using PyMOL (The PyMOL Molecular Graphics System, Version 2.3 Schrödinger, LLC).

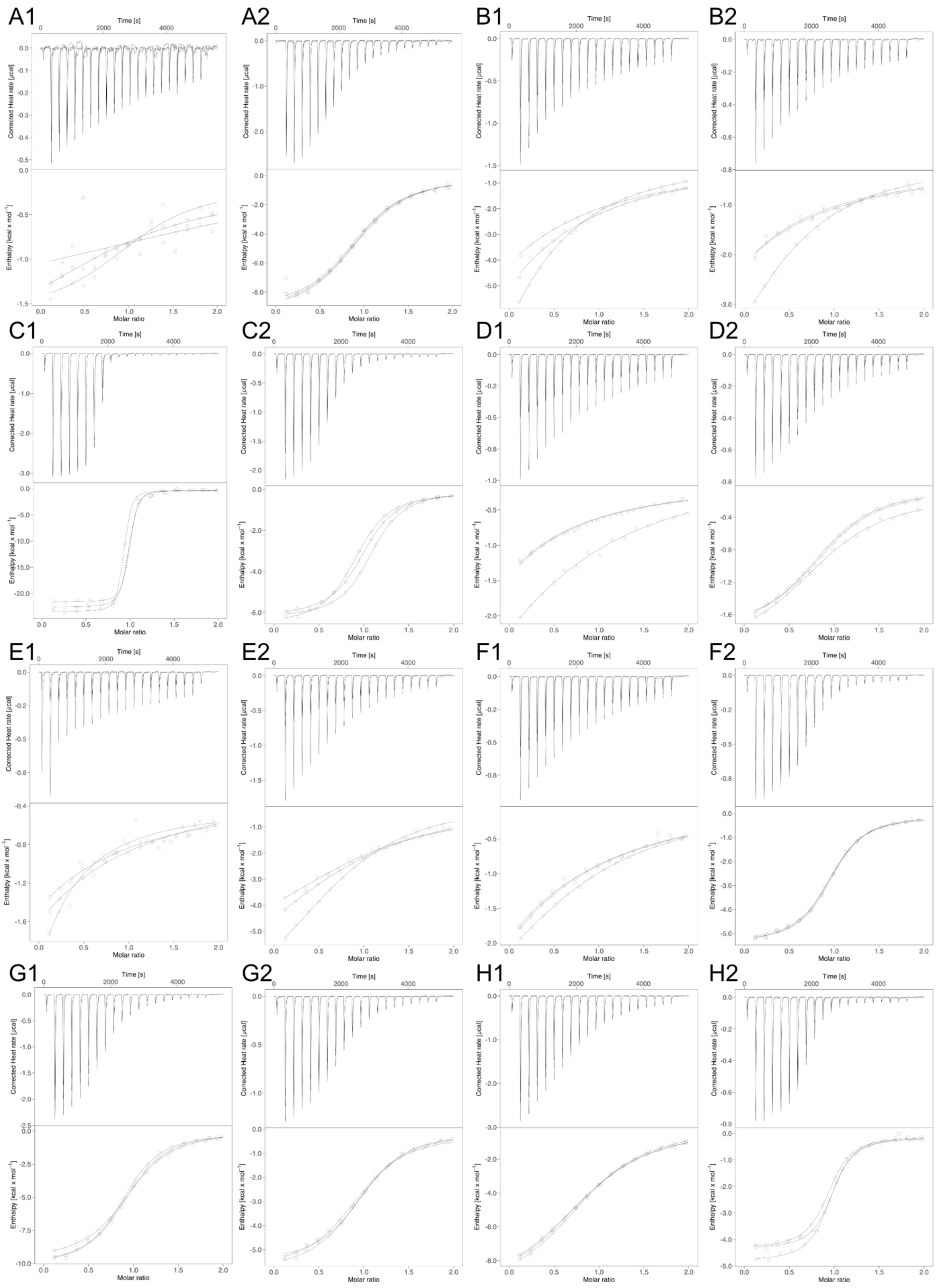

Figure S2a: ITC measurements and analysis related to Table 1. Measurements with PUT are labelled with 1 and measurements with SPD are labelled with 2. A: PotF/D, B: PotF_Prox, C: PotF_Abox, D: PotF_Dist, E: PotF_Abox_Prox, F: PotF_Abox_Dist, G: PotF_Abox-S87Y, H: PotF_Abox-A182D. Plots contain data from 3 biological replicates.

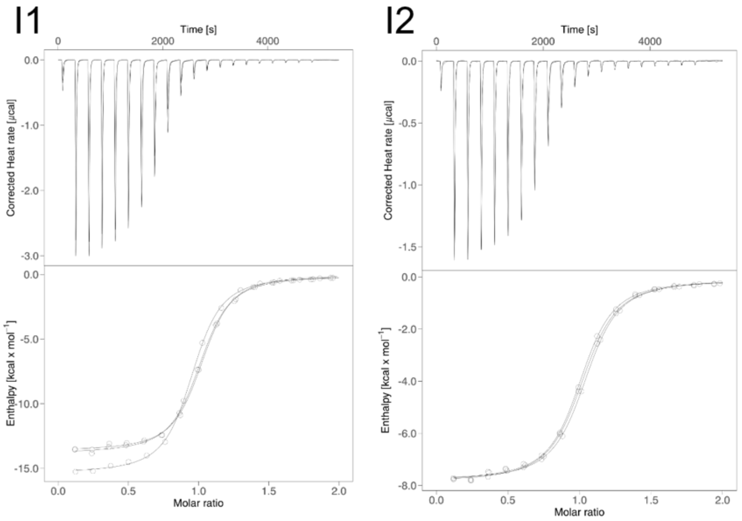

Figure S2b: ITC measurements and analysis related to Table 1. Measurements with PUT are labelled with 1 and measurements with SPD are labelled with 2. I: PotF_Abox-L348Q. Plots contain data from 3 biological replicates.

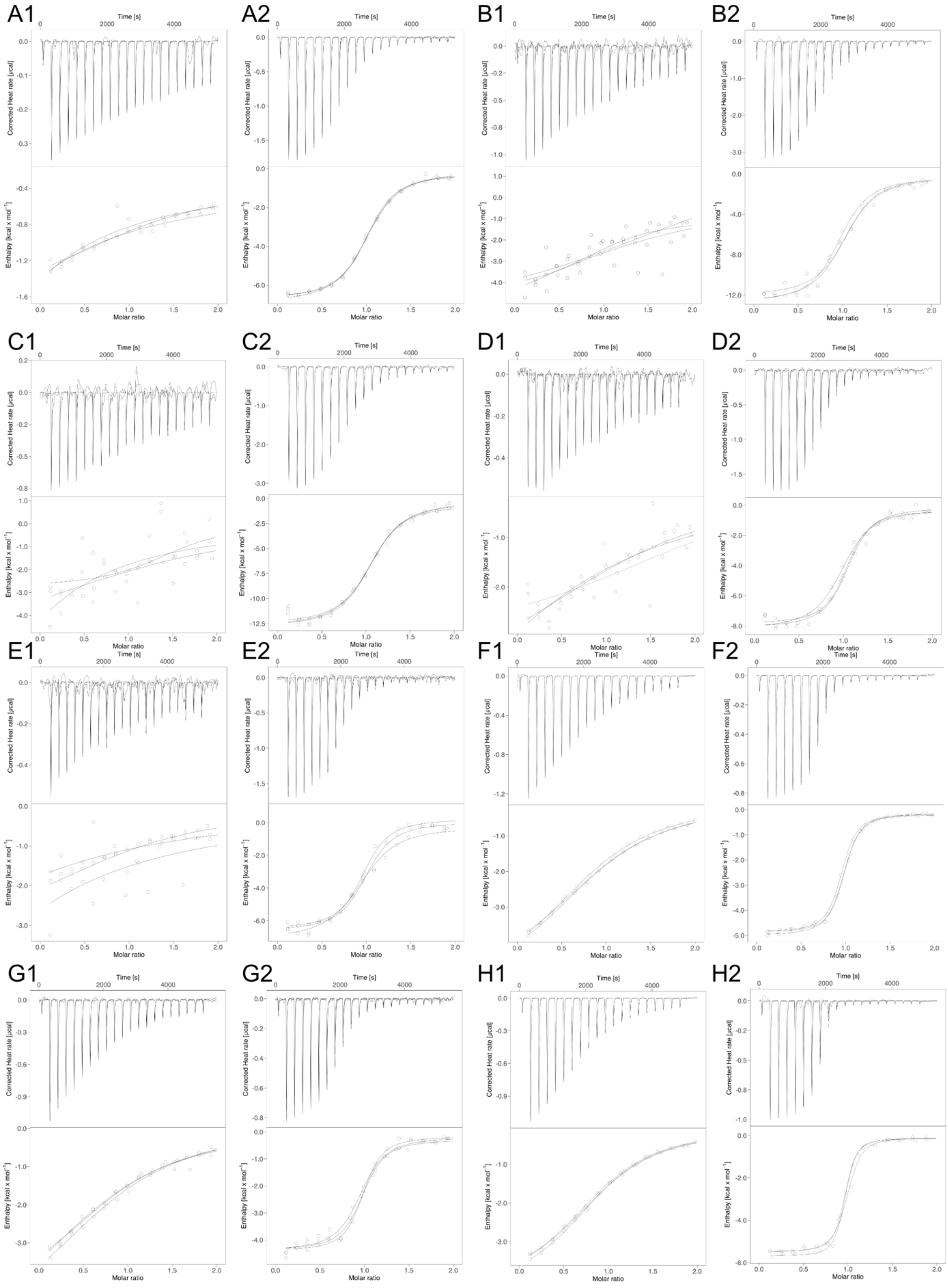

Figure S3: ITC measurements and analysis related to Table 2. Measurements with PUT are labelled with 1 and measurements with SPD are labelled with 2. A: PotF/D-E39D-Y87S, B: PotF/D-E39D-F88A, C: PotF/D-E39D-F88L, D: PotF/D-E39D-Y87S-F88Y, E: PotF/D-S247D, F: PotF/D-E39D-F88A-S247D, G: PotF/D-E39D-F88L-S247D, H: PotF/D-E39D-Y87S-F88Y-S247D. Plots contain data from 3 biological replicates.

Table S1: Residue groups in PotF and their respective counterparts in PotD. All resulting mutations in PotF/D are listed as well.

	PotF	PotD	Mutations in PotF/D
**Proximal (Prox)**	S38, D39 & D247	T35, E39 & S232	S38T, D39E & D247S
**Aromatic box (Abox)**	W37, W244 & F276	W34, W229 & W255	F276W
**Distal (Dist)**	S85, S87, A182, E285, D278 & L348	S83, Y85, D168, E171, D257 & Q327	S87Y, A182D & L348Q

Table S2: RMS calculated by aligning the stated residues from PotF/D-E39D-F88A-S247D onto PotF/D-E39D-F88L-S247 via PyMol without outlier rejection cycles.

**Residues** *Align F88A onto L*	**RMS over Cα Chain A/A**	**RMS over Cα Chain A/B**	**RMS over Cα Chain B/A**	**RMS over Cα Chain B/B**
29-369	0.481	0.478	0.370	0.409
29-85	0.272	0.259	0.179	0.190
86-123	0.610	0.544	0.518	0.472
124-369	0.335	0.376	0.321	0.371

Table S3: Crystallographic data and refinement statistics for all solved crystal structures.

	PotF/D	E39D-F88L	E39D-Y87S	E39D-Y87S-F88Y	S247D	E39D-F88L-S247D	E39D-F88A-S247D	E39D-Y87S-F88Y-S247D
**PDB ID**	7OYZ	7OYT	7OYS	7OYU	7OYY	7OYW	7OYV	7OYX
**Data collection**								
Wavelength [Å]	1.0370	0.9184	0.9184	0.9184	0.9184	0.9184	0.9184	0.9184
Resolution range [Å]	46.14 - 1.49 (1.54 - 1.49)	39.01 - 1.60 (1.68 - 1.60)	39.29 - 1.57 (1.63 - 1.57)	46.36 - 1.95 (2.02 - 1.95)	35.15 - 1.36 (1.41 - 1.36)	49.39 - 1.28 (1.33 - 1.28)	47.58 - 1.90 (1.97 - 1.90)	40.70 - 1.37 (1.41 - 1.37)
Space group	$P\ 2_1\ 2_1\ 2_1$	$P\ 2_1\ 2_1\ 2_1$	$P\ 2_1\ 2_1\ 2_1$	$P\ 2_1\ 2_1\ 2_1$	$P\ 2_1\ 2_1\ 2_1$	$P\ 2_1\ 2_1\ 2$	$C\ 2\ 2\ 2_1$	$P\ 2_1\ 2_1\ 2$
Cell parameter								
a, b, c, [Å]	37.20 81.85 111.74	37.14 78.03 118.44	37.26 78.58 114.44	37.14 78.61 114.83	37.06 82.15 111.07	116.88 71.29 92.42	73.61 122.58 190.32	117.16 71.49 92.66
α,β,γ [°]	90.0, 90.0, 90.0	90.0, 90.0, 90.0	90.0, 90.0, 90.0	90.0, 90.0, 90.0	90.0, 90.0, 90.0	90.0, 90.0, 90.0	90.0, 90.0, 90.0	90.0, 90.0, 90.0
Total reflections	720880 (67643)	292090 (26997)	260238 (25374)	138481 (14023)	538447 (52254)	1306722 (114299)	594419 (40596)	1212027 (117504)
Unique reflections	56881 (5388)	46390 (4363)	47693 (4677)	25021 (2455)	73425 (7123)	198363 (19073)	62244 (5363)	163742 (16029)
Multiplicity	12.7 (12.5)	6.5 (6.2)	5.5 (5.4)	5.5 (5.7)	7.3 (7.3)	6.6 (6.0)	9.5 (7.6)	7.4 (7.3)
Completeness [%]	99.6 (95.7)	99.8 (99.7)	99.6 (99.5)	98.9 (99.2)	99.4 (96.2)	99.3 (96.4)	90.9 (79.5)	99.8 (98.9)
Mean I/sigma [I]	11.11 (0.55)	9.03 (0.66)	10.35 (0.73)	6.44 (0.79)	7.58 (0.61)	8.80 (0.59)	13.74 (1.00)	7.21 (0.46)
Wilson B-factor	23.7	23.3	21.8	30.1	14.5	16.3	33.1	16.6
No. of molecules per a.u.	1	1	1	1	1	2	2	2
Matthews coefficient	2.16	2.26	2.13	2.14	2.15	2.53	2.82	2.55
R_{merge}	0.136 (4.220)	0.128 (2.421)	0.098 (2.027)	0.206 (2.231)	0.171 (2.530)	0.104 (2.316)	0.121 (2.003)	0.154 (3.290)
R_{meas}	0.142 (4.397)	0.139 (2.645)	0.108 (2.242)	0.227 (2.451)	0.184 (2.721)	0.114 (2.536)	0.128 (2.139)	0.166 (3.540)
R_{pim}	0.040 (1.218)	0.054 (1.051)	0.045 (0.943)	0.093 (0.996)	0.068 (0.988)	0.044 (1.015)	0.039 (0.712)	0.061 (1.295)
$CC_{1/2}$	0.999 (0.281)	0.998 (0.273)	0.999 (0.256)	0.994 (0.262)	0.997 (0.280)	0.997 (0.233)	0.998 (0.375)	0.997 (0.240)
CC*	1.000 (0.662)	0.999 (0.655)	1.000 (0.639)	0.998 (0.644)	0.999 (0.661)	0.999 (0.615)	1.000 (0.739)	0.999 (0.622)
**Refinement**								

Reflections used in refinement	56879 (5388)	46390 (4363)	47683 (4677)	25007 (2581)	73415 (4555)	198355 (19073)	62236 (5363)	163728 (16025)
Reflections used for R_{free}	2101 (199)	2100 (197)	2098 (206)	1251 (135)	2099 (134)	2098 (202)	2101 (182)	2099 (206)
R_{work}	0.177 (0.425)	0.178 (0.362)	0.174 (0.340)	0.201 (0.320)	0.156 (0.334)	0.132 (0.332)	0.227 (0.403)	0.168 (0.416)
R_{free}	0.216 (0.446)	0.209 (0.380)	0.209 (0.345)	0.242 (0.355)	0.186 (0.367)	0.161 (0.346)	0.276 (0.459)	0.194 (0.461)
CC_{work}	0.958 (0.614)	0.965 (0.627)	0.966 (0.587)	0.963 (0.606)	0.974 (0.635)	0.978 (0.577)	0.946 (0.578)	0.978 (0.559)
CC_{free}	0.967 (0.495)	0.961 (0.646)	0.955 (0.635)	0.950 (0.493)	0.975 (0.699)	0.970 (0.438)	0.901 (0.630)	0.976 (0.522)
Number of non-hydrogen atoms	3106	3078	3155	2964	3444	6970	5749	6639
macromolecules	2750	2726	2764	2729	2855	5994	5432	5718
solvent	341	218	301	180	545	878	230	814
Protein residues	341	341	342	341	342	696	682	694
RMS bond lengths [Å]	0.002	0.011	0.005	0.005	0.007	0.008	0.007	0.017
RMS bond angles [°]	0.52	1.05	0.75	0.69	0.91	1.05	0.86	1.46
Ramachandran favored [%]	98.2	97.9	98.2	97.6	97.9	97.8	97.5	98.1
Ramachandran allowed [%]	1.8	2.1	1.8	2.4	2.1	2.2	2.5	1.9
Ramachandran outliers [%]	0.0	0.0	0.0	0.0	0.0	0.0	0.0	0.0
Rotamer outliers [%]	0.7	0.7	1.0	0.7	1.3	1.8	1.0	1.0
Clashscore	1.27	5.03	2.61	4.85	2.39	6.09	6.49	3.54
Average B-factor	31.14	31.4	28.3	35.9	19.7	21.8	37.4	23.5
macromolecules	29.7	29.6	26.8	35.3	16.9	19.2	37.3	21.7
solvent	42.1	40.9	36.8	40.6	35.0	37.8	36.8	33.4
Number of TLS groups		1	1	1			2	2

Table S4: Oligonucleotides used for the generation of PotF and PotF/D variants via QuickChange during this study

Mutation	Orientation	Sequence (5'-3')
S38T & D39E	forward	TTTATAACTGGACCGAATATATCGCCCCG
	reverse	CGGGGCGATATATTCGGTCCAGTTATAAA
E39D	forward	CATTTATAACTGGACTGATTATATCGCCCCGGACACG
	reverse	CGTGTCCGGGGCGATATAATCAGTCCAGTTATAA
S87Y	forward	GGTTCCATCTGCCTACTTTCTGGAGCGCC
	reverse	GGCGCTCCAGAAAGTAGGCAGATGGAACC
Y87S	forward	GGTGGTTCCATCTGCCAGCTTTCTGGAGCGCCAG
	reverse	CTGGCGCTCCAGAAAGCTGGCAGATGGAACCACC
F88A	forward	GTGGTTCCATCTGCCTACGCGCTGGAGCGCCAGTTGACT
	reverse	AGTCAACTGGCGCTCCAGCGCGTAGGCAGATGGAACCAC
F88L	forward	GGTTCCATCTGCCTACTTACTGGAGCGCC
	reverse	GGCGCTCCAGTAAGTAGGCAGATGGAACC
F88Y	forward	TTCCATCTGCCAGCTATCTGGAGCGCCAG
	reverse	CTGGCGCTCCAGATAGCTGGCAGATGGAA
A182D	forward	CTCTTTCCTGGATGATCCAGAAGAAGTTT
	reverse	AAACTTCTTCTGGATCATCCAGGAAAGAG
D247S	forward	CGGCTGGGCAGGTTCTGTCTGGCAGGCGT
	reverse	ACGCCTGCCAGACAGAACCTGCCCAGCCG
S247D	forward	ATCGGCTGGGCAGGTGATGTCTGGCAGGCG
	reverse	CGCCTGCCAGACATCACCTGCCCAGCCGAT
F276W	forward	AGGGGCGATGGCGTGGTTTGATGTATTCG
	reverse	CGAATACATCAAACCACGCCATCGCCCCT
L348Q	forward	AAGCTGTTCACTCAGAAAGTGCAGGATCC
	reverse	GGATCCTGCACTTTCTGAGTGAACAGCTT

Table S5: Concentrations of protein and ligand solutions used for the triplicate ITC measurements during this study

Protein Variant	Protein concentrations [µM]	Ligand	Ligand concentrations [mM]
PotF_Prox	***1)*** 348 ***2)*** 360 ***3)*** 261	PUT	***1)*** 3.28 ***2)*** 3.64 ***3)*** 2.70
	1) 348 ***2)*** 235 ***3)*** 261	SPD	***1)*** 3.28 ***2)*** 2.40 ***3)*** 2.70
PotF_Abox	***1)*** 146 ***2)*** 147 ***3)*** 140	PUT	***1)*** 1.16 ***2)*** 1.66 ***3)*** 1.53
	1) 328 ***2)*** 298 ***3)*** 283	SPD	***1)*** 4.31 ***2)*** 3.27 ***3)*** 3.09
PotF_Dist	***1)*** 588 ***2)*** 502 ***3)*** 472	PUT	***1)*** 6.00 ***2)*** 5.00 ***3)*** 5.00
	1) 602 ***2)*** 502 ***3)*** 470	SPD	***1)*** 6.00 ***2)*** 5.00 ***3)*** 5.00
PotF_Abox_Prox	***1)*** 425 ***2)*** 208 ***3)*** 209	PUT	***1)*** 4.25 ***2)*** 2.00 ***3)*** 2.10
	1) 409 ***2)*** 205 ***3)*** 209	SPD	***1)*** 4.25 ***2)*** 2.00 ***3)*** 2.10
PotF_Abox_Dist	***1)*** 563 ***2)*** 382 ***3)*** 425	PUT	***1)*** 6.00 ***2)*** 3.80 ***3)*** 4.25
	1) 194 ***2)*** 200 ***3)*** 209	SPD	***1)*** 2.00 ***2)*** 2.00 ***3)*** 2.09
PotF_Abox_S87Y	***1)*** 235 ***2)*** 250 ***3)*** 291	PUT	***1)*** 2.35 ***2)*** 2.55 ***3)*** 3.00
	1) 235 ***2)*** 250 ***3)*** 291	SPD	***1)*** 2.35 ***2)*** 2.55 ***3)*** 3.00
PotF_Abox_A182 D	***1)*** 381 ***2)*** 433 ***3)*** 345	PUT	***1)*** 3.80 ***2)*** 4.40 ***3)*** 3.50
	1) 193 ***2)*** 217.5 ***3)*** 161	SPD	***1)*** 2.00 ***2)*** 2.20 ***3)*** 1.77
PotF_Abox_L348Q	***1)*** 240 ***2)*** 238 ***3)*** 227	PUT	***1)*** 2.40 ***2)*** 2.40 ***3)*** 2.35
	1) 240 ***2)*** 235 ***3)*** 245	SPD	***1)*** 2.40 ***2)*** 2.40 ***3)*** 2.45
PotF/D	***1)*** 470 ***2)*** 370 ***3)*** 360	PUT	***1)*** 4.70 ***2)*** 3.70 ***3)*** 3.60
	1) 395 ***2)*** 370 ***3)*** 360	SPD	***1)*** 4.00 ***2)*** 3.70 ***3)*** 3.60
PotF/D-E39D-Y87S	***1)*** 319 ***2)*** 308 ***3)*** 312	PUT	***1)*** 3.20 ***2)*** 3.10 ***3)*** 3.10
	1) 319 ***2)*** 308 ***3)*** 312	SPD	***1)*** 3.20 ***2)*** 3.10 ***3)*** 3.10
PotF/D-E39D-F88A	***1)*** 308 ***2)*** 317 ***3)*** 310	PUT	***1)*** 3.00 ***2)*** 3.10 ***3)*** 3.10
	1) 308 ***2)*** 317 ***3)*** 310	SPD	***1)*** 3.00 ***2)*** 3.10 ***3)*** 3.10
PotF/D-E39D-F88L	***1)*** 294 ***2)*** 296 ***3)*** 299	PUT	***1)*** 2.90 ***2)*** 2.90 ***3)*** 3.00
	1) 294 ***2)*** 300 ***3)*** 299	SPD	***1)*** 2.90 ***2)*** 3.00 ***3)*** 3.00
PotF/D-E39D-Y87S-F88Y	***1)*** 260 ***2)*** 265 ***3)*** 248	PUT	***1)*** 2.60 ***2)*** 2.60 ***3)*** 2.50
	1) 260 ***2)*** 265 ***3)*** 248	SPD	***1)*** 2.60 ***2)*** 2.60 ***3)*** 2.50
PotF/D-S247D	***1)*** 310 ***2)*** 305 ***3)*** 305	PUT	***1)*** 3.00 ***2)*** 3.00 ***3)*** 3.00
	1) 310 ***2)***305 ***3)*** 305	SPD	***1)*** 3.00 ***2)*** 3.00 ***3)*** 3.00
PotF/D-E39D-F88A-S247D	***1)*** 361 ***2)*** 378 ***3)*** 374	PUT	***1)*** 4.00 ***2)*** 4.00 ***3)*** 4.00
	1) 183 ***2)*** 193 ***3)*** 189	SPD	***1)*** 2.00 ***2)*** 2.00 ***3)*** 2.00
PotF/D-E39D-F88L- S247D	***1)*** 391 ***2)*** 360 ***3)*** 370	PUT	***1)*** 4.00 ***2)*** 3.60 ***3)*** 3.70
	1) 205 ***2)*** 180 ***3)*** 220	SPD	***1)*** 2.00 ***2)*** 1.80 ***3)*** 2.20
PotF/D-E39D-Y87S-F88Y- S247D	***1)*** 393 ***2)*** 388 ***3)*** 379	PUT	***1)*** 4.00 ***2)*** 4.00 ***3)*** 4.00
	1) 202 ***2)*** 186 ***3)*** 189	SPD	***1)*** 2.10 ***2)*** 2.10 ***3)*** 2.00

Table S6: Full crystallization conditions, protein concentrations and cryogenic solutions used for the structure determination of all constructs throughout this study.

Construct	Concentration	Condition	Cryogenic Solution
PotFD-E39D-Y87S	15 mg/ml	0.085 M Sodium acetate pH 4.7, 0.17M Ammonium acetate, 32.5% PEG 4000, 15% Glycerol	-
PotFD-E39D-F88L	30 mg/ml	0.1 M Sodium acetate pH 4.6, 0.2 M Ammonium acetate, 30% PEG 4000	25% Glycerol + SPD
PotFD-E39D-Y87S-F88Y	40 mg/ml	0.1 M Sodium acetate pH 4.6, 0.2 M Ammonium acetate, 30% PEG 4000	25% Glycerol + SPD
PotFD-E39D-F88A-S247D	30 mg/ml	2.4 M AmSO4, 0.1 M Bicine pH 8.3, 4.5% Jeffamine M600	50% 3.4 M Malonate pH 8.3 + SPD
PotFD-E39D-F88L-S247D	40 mg/ml	2.4 M AmSO4, 0.1 M Bicine pH 9.0, 5% Jeffamine M600	50% 3.4 M Malonate pH 8.8 + SPD
PotFD-E39D-Y87S-F88Y-S247D	30 mg/ml	2.4 M AmSO4, 0.1 M Bicine pH 8.3, 4.5% Jeffamine M600	50% 3.4 M Malonate pH 8.3 + SPD
PotFD-S247D	40 mg/ml	0.1 M MES pH 5, 30% PEG 6000	25% Glycerol + SPD
